# 805. Influence of Magnetic Field Arrangements on Cultured Epidermal Autografts Growth Derived from a Modified Technique

**DOI:** 10.1093/jbcr/irag033.183

**Published:** 2026-04-06

**Authors:** Wayne George Kleintjes, Tarryn Kay Prinsloo

**Affiliations:** Sheik Khalifa Hospital Fujairah, Cape Town, Western Cape; Cape Peninsula University of Technology, Cape Town, Western Cape

## Abstract

**Introduction:**

Cellular electric fields (EFs) influence key mechanisms that regulate cellular behaviors such as migration, proliferation, and growth. Varying magnetic fields (MFs) may influence these EF-mediated processes in cultured epidermal autografts (CEA). Using a modified culture technique, this pilot study explored whether different MF arrangements affect CEA growth. We hypothesized that continuous MF exposure would promote greater cell growth and pigmentation compared with interrupted fields.

**Methods:**

Epidermal fragments were isolated from skin biopsies, and the keratinocytes enzymatically retrieved with trypsin were seeded onto routinely used wound dressings with bacteria- and fungi-binding properties. The keratinocytes were supplemented with autologous platelet-rich plasma (PRP) and placed directly on varying magnet arrangements with relatively low MF strength. Incubation occurred in pediatric incubators at 37°C for 1 week. CEA were supplemented daily with fresh PRP and every 3–4 days with hydrogel. Light microscopy (100X) was used to visually assess growth progression in terms of viscosity, density, and pigmentation as primary outcomes.

**Results:**

Overall, CEA cell growth was more viscous and denser with darker pigmentation on the dressing sheets that had magnetic arrangements with continuous MFs compared to those arrangements with gaps.

**Conclusions:**

CEA cultivated under continuous MFs exhibited more favorable growth characteristics, suggesting that even low-strength MFs can influence EF-mediated proliferation. These pilot findings support the potential role of MF stimulation in enhancing CEA growth and warrant further investigation for validation and mechanistic insights.

**Applicability of Research to Practice:**

If confirmed in larger studies, MF stimulation could provide a simple, low-cost adjunct to optimize CEA growth in clinical and laboratory settings. This may improve graft availability, reduce culture times, and enhance outcomes for patients requiring epidermal reconstruction, particularly in resource-limited contexts.

**Funding for the study:**

N/A.

